# T-cell counts in peripheral blood at leukapheresis predict responses to subsequent CAR-T cell therapy

**DOI:** 10.1038/s41598-022-23589-9

**Published:** 2022-11-04

**Authors:** Fumiya Wada, Tomoyasu Jo, Yasuyuki Arai, Toshio Kitawaki, Chisaki Mizumoto, Junya Kanda, Momoko Nishikori, Kouhei Yamashita, Miki Nagao, Akifumi Takaori-Kondo

**Affiliations:** 1 Department of Hematology and Oncology, Graduate School of Medicine, Kyoto University, Kyoto, Japan; 2 Department of Clinical Laboratory Medicine, Graduate School of Medicine, Kyoto University, Kyoto, Japan; 3 Center for Research and Application of Cellular Therapy, Kyoto University Hospital, 54 Shogoin Kawahara-cho, Sakyo-ku, Kyoto, 606-8507 Japan

**Keywords:** B-cell lymphoma, Outcomes research

## Abstract

Prediction of responses to chimeric antigen receptor (CAR)-T cell therapies is essential to maximize their therapeutic efficacy for diffuse large B-cell lymphoma (DLBCL). While several tumor-intrinsic risk factors of resistance and/or early relapse have been identified, clinically useful markers that determine potential activity of CAR-T cells have not been fully investigated. T-cell property at the time of leukapheresis may serve as such a marker. Therefore, we evaluated the clinical impact of CD3^+^ cell count in peripheral blood at leukapheresis on clinical outcomes of CAR-T cell therapy. In total, 44 patients with relapsed or refractory (r/r) DLBCL who received tisagenlecleucel at Kyoto University Hospital were included. According to CD3^+^ cell counts, patients were categorized into CD3^LOW^ and CD3^HIGH^ groups with a threshold of 553/μL, based on receiver operating characteristic curve analysis. 1-year progression-free survival was significantly higher in the CD3^HIGH^ group than the CD3^LOW^ group (68.3% vs. 17.3%; adjusted hazard ratio [aHR], 0.37; p = 0.042). Overall survival was also superior in the CD3^HIGH^ group (aHR, 0.24; p = 0.043). Moreover, higher CD3^+^ cell counts at leukapheresis were associated with significantly higher lymphocyte counts in peripheral blood at day 7 after CAR-T cell infusion (median 860 vs. 420/μL, P = 0.021), suggesting more extensive expansion of infused CAR-T cells in vivo. In conclusion, we demonstrated that the CD3^+^ cell count at leukapheresis predicts both expansion of CAR-T cells after infusion and outcomes of CAR-T cell therapy, and are useful for building comprehensive therapeutic strategies at the time of leukapheresis.

## Introduction

Diffuse large B cell lymphoma (DLBCL) is curable with standard anthracycline-based chemotherapy regimens, but patients with relapsed or refractory (r/r) DLBCL still have dismal prognosis, even with intensified chemotherapy and autologous or allogeneic stem cell transplantation^1^. CD19-directed chimeric antigen receptor (CAR)-T cell therapies have remarkably improved prognosis for a subset of patients with r/r DLBCL^2^. While responders to CAR-T cell therapies have promising prognosis, those with incomplete responses have grim prognosis. Moreover, even in patients who once responded to CD19-directed CAR-T therapies, about 20% are reported to experience relapses later^3–5^. Therefore, a risk-stratified approach, based on early identification of high-risk patients for resistance or relapse to CAR-T cell therapies, are required to optimize them.

Recently, several prognostic clinical factors for CAR-T cell therapy, mostly focused on patient characteristics or disease status, have been reported. For instance, high tumor volume at infusion is associated with decreased efficacy of CAR-T cell therapy, which suggests that disease control before administration of CAR-T cells is important for better responses^6,7^. Certain genetic alterations, such as TP53 mutations, have been reported as tumor-intrinsic biomarkers that inform inferior responses^8^. Such parameters on the tumor side have been rapidly established. However, efficacy of CAR-T cell therapy is determined not only by tumor-intrinsic factors, but also by the parameters on the CAR-T cell side and the latter have not been fully analyzed^9^. Moreover, it is essential to predict efficacy of CAR-T cell therapies as early as possible, i.e., at leukapheresis, and to optimize treatment strategy before and after therapies in order to maximize their effects, because candidates for CAR-T cell therapy often have highly refractory, progressive disease, as well as frequent complications.

Therefore, in this study, we focused on the prognostic value of CD3^+^ cell count in peripheral blood at leukapheresis on outcomes of CAR-T cell therapy. Our findings provide a novel biomarker that enables us to predict responses as early as the time of leukapheresis. This marker should help to improve outcomes for CAR-T eligible patients with r/r DLBCL.

## Patients and methods

### Study cohort

This study was performed by analysing all consecutive patients with r/r DLBCL who received tisagenlecleucel at Kyoto University Hospital, Kyoto Japan, between December 2019 and October 2021. Follow-up time was fixed in December 2021. The diagnosis of DLBCL was based on the WHO classification of tumors of hematopoietic and lymphoid tissues (revised 4th edition)^10^. All patients were relapsed or refractory to two or more prior lines of therapy. This study was approved by the Ethics Committee of Kyoto University, and was conducted in accordance with the principles of the Declaration of Helsinki. Informed consent was obtained from all the patients.

### Endpoints and variables

The primary endpoint of this study was progression‐free survival (PFS). Overall survival (OS) was evaluated as a secondary endpoint. PFS was defined as the time from the date of CAR-T infusion to the date of documented disease progression, relapse, death, or the last date of follow‐up. OS was calculated from the date of CAR-T infusion to the last date of follow‐up or death. Disease status at leukapheresis, at infusion, and after infusion was assessed using the Revised Response Criteria for Malignant Lymphoma^11^. Progression of relapse was defined based on morphological and clinical evidence of disease activity. All variables shown in tables and figures were retrospectively obtained from patient records. To simplify risk stratification, we divided our patients into two risk groups (CD3^LOW^ and CD3^HIGH^ group) by employing the receiver operating characteristic (ROC) curve of CD3^+^ cell count at leukapheresis for progression-free survival (PFS) at 1 year.

### Statistical analysis

Continuous variables were summarized using medians and ranges, and categorical variables were summarized as counts and percentages. For comparisons between groups, patients and disease characteristics were compared using the Mann‐Whitney U test for continuous variables and Fisher’s exact test for categorical variables. Correlation between CD3^+^ cell count at leukapheresis and lymphocyte count after infusion were analyzed using Pearson’s correlation. Probabilities of PFS and OS were estimated using the Kaplan‐Meier method, and were compared between groups using the log‐rank test. In multivariate analysis, Cox proportional hazards models were used to evaluate the effects of CD3^+^ cell count in peripheral blood at leukapheresis on PFS and OS in combination with other clinically relevant variables, including prior lines of chemotherapies at leukapheresis (0–3 or > 3) and disease status at leukapheresis (complete response [CR]/partial response [PR] or stable disease [SD]/progressive disease [PD]). Statistical significance was set at p < 0.05. All statistical analyses were performed using R (version 3.1.1; R Development Core Team) and EZR (Saitama Medical Center, Jichi Medical University, Saitama, Japan)^12^.

## Results

### Patient characteristics

In total, 44 patients were included in this study (Table 1). The study cohort comprised 23 females and 21 males, with a median age of 60 years (range, 20–73 years). The median CD3^+^ cell count in peripheral blood at leukapheresis was 687/μL (range, 60–2234). According to the ROC curve of CD3^+^ cell count for PFS at 1 year, the cut off value was set at 553/μL. Then, we defined patients with CD3^+^ cell counts ≤ 553/μL as the CD3^LOW^ group (20 patients), and those with higher CD3^+^ cell counts as the CD3^HIGH^ group (24 patients). There was no significant difference between the two groups in age at infusion. In terms of DLBCL characteristics, the proportions of patients with germinal center B-cell-like (GCB) DLBCL, double-expressor lymphoma (DEL), transformed follicular lymphoma (tFL) were comparable between the two groups (P = 0.545, 0.475, and 0.734, respectively). There were no significant differences between groups in prior lines of chemotherapy at leukapheresis, or in disease status at leukapheresis, or infusion. Time from diagnosis to leukapheresis was significantly longer in the CD3^HIGH^ group compared with the CD3^LOW^ group (P = 0.036). Lymphocyte depletion protocol was composed of fludarabine with cyclophosphamide (Flu/Cy, n = 37) and bendamustine-based regimens (n = 5), and was not administered in two patients with exceptionally low lymphocyte counts. The characteristics of CAR-T cell products in each group are shown in Supplementary Table 1. The occurrence or grade of cytokine release syndrome (CRS) and the use of tocilizumab in each group are shown in Supplementary Table 2. We used tocilizumab if patients had continuous high fever refractory to conservative treatment or developed grade 2 CRS.

**Table 1:** Patient characteristics.

	Total (n = 44)	CD3^LOW^ (n = 20)	CD3^HIGH^ (n = 24)	P
**Sex**				0.382
Female	23 (52.3)	12 (60.0)	11 (45.8)	
Male	21 (47.7)	8 (40.0)	13 (54.2)	
**Age at infusion**				0.109
Median, range	60 (20–73)	58 (20–69)	63 (27–73)	
**Hans**				0.545
GCB	23 (52.3)	9 (5.0)	14 (58.3)	
Non-GCB	21 (47.7)	11 (55.0)	10 (41.7)	
**Double expressor**				0.673
Yes	12 (27.3)	7 (35.0)	5 (20.8)	
No	22 (50.0)	9 (45.0)	13 (54.2)	
Missing	10 (22.7)	4 (20.0)	6(25.0)	
**Transformed follicular lymphoma**				0.734
Yes	10 (22.7)	4 (20.0)	6 (25.0)	
No	34 (77.3)	16 (80.0)	18 (75.0)	
**Prior lines at leukapheresis**				0.547
0–3	18 (40.9)	7 (35.0)	11 (45.8)	
> 3	26 (59.1)	13 (65.0)	13 (54.2)	
**Disease status at leukapheresis**				0.519
CR/PR	14 (31.8)	5 (25.0)	9 (37.5)	
SD/PD	30 (68.2)	15 (75.0)	15 (62.5)	
**CD3**^**+**^** cell count at leukapheresis (/μL)**				< 0.001
Median	687.3	304.6	971.7	
Range	59.9–2234.0	59.8–553.0	632.5–2234.0	
**LDH at leukapheresis**^**a**^** (IU/L)**				0.294
Median	224	203	228	
Range	138–613	138–498	168–613	
**Time from diagnosis to leukapheresis (days)**				0.036
Median	483	401.5	658	
Range	61–3331	61–2746	205–3331	
**Lymphocyte depletion protocol**				0.673
Flu/Cy	37 (84.1)	18 (90.0)	19 (79.2)	
Bendamustine-based	5 (11.4)	1 (5.0)	4 (16.7)	
Omitted	2 (4.5)	1 (5.0)	1 (4.2)	
**Disease status at infusion**				0.111
CR/PR	15 (34.1)	4 (20.0)	11 (45.8)	
SD/PD	29 (65.9)	16 (0.0)	13 (54.2)	

*GCB* germinal center B-cell-like, *CR* complete response, *PR* partial response, *SD* stable disease, *PD* progressive disease.

^a^Normal range; 124 to 222 IU/L.

### OS and PFS according to the CD3^+^ cell count at leukapheresis

**Figure 1:**
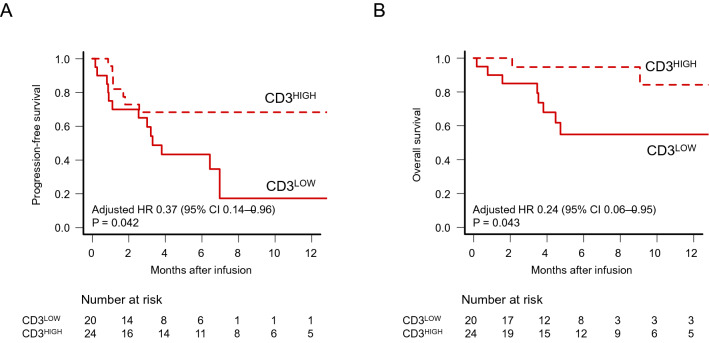
Effects of CD3^+^ cell counts in peripheral blood at leukapheresis on survival. Kaplan–Meier estimates of progression-free survival (PFS) (**A**) and overall survival (OS) (**B**) according to the number of CD3^+^cells at leukapheresis.

We compared effects of CD3^+^ cell count at leukapheresis on survival after CAR-T cell infusion, and found that the 1‐year PFS was significantly lower in the CD3^LOW^ group (17.3%, 95%CI 1.50–48.3%) than in the CD3^HIGH^ group (68.3%, 95%CI 44.8–83.5%; p = 0.032) (Fig. 1A). After adjusting prior lines at leukapheresis (0–3 or > 3) and disease status at leukapheresis (CR/PR or SD/PD) as covariates, the adjusted hazard ratio (aHR) of PFS was 0.37 (95% CI: 0.14–0.96, p = 0.042) for the CD3^HIGH^ group compared to the CD3^LOW^ group (Table 2). When CD3^+^ cell count was treated as a continuous variable, aHR was 0.90 (95% CI: 0.81–1.00, p = 0.052) for every 100/μL increase.

**Table 2:** Multivariate analysis of factors affecting PFS and OS.

Outcome	Variables		HR (95%CI)	P
**PFS**	CD3^+^ cells at leukapheresis	Low	1.00	Reference
		High	0.37 (0.14–0.96)	0.042
	Prior lines	0–3	1.00	Reference
		> 3	0.78 (0.32–1.91)	0.588
	Disease status	CR/PR	1.00	Reference
		SD/PD	0.90 (0.32–2.51)	0.834
**OS**	CD3^+^ cells at leukapheresis	Low	1.00	Reference
		High	0.24 (0.06–0.95)	0.043
	Prior lines	0–3	1.00	Reference
		> 3	0.62 (0.17–2.30)	0.476
	Disease status	CR/PR	1.00	Reference
		SD/PD	1.86 (0.51–6.82)	0.347

*PFS* progression‐free survival, *OS* overall-survival, *CR* complete response, *PR* partial response, *SD* stable disease, *PD* progressive disease.

Univariate analysis for OS showed a trend for worse 1‐year OS in the CD3^LOW^ group (54.9%, 95% CI 29.2–74.7%) compared to the CD3^HIGH^ group (84.2%, 95% CI 47.0–96.2%; p = 0.054) (Fig. 1B). Multivariate analysis revealed that lower CD3^+^ cell counts significantly reduced OS (Table 2). aHR for OS in the CD3^HIGH^ group versus the CD3^LOW^ group was 0.24 (95% CI 0.06–0.95, p = 0.043) (Table 2). When CD3^+^ cell count was treated as a continuous variable, aHR was 0.88 (95% CI 0.75–1.03, p = 0.102) for every 100/μL increase.

**Figure 2:**
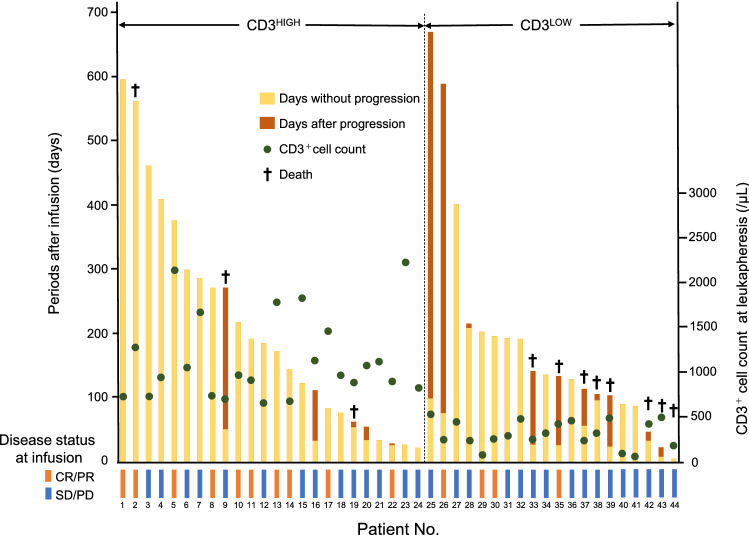
Clinical course of patients according to CD3^+^ cell counts at leukapheresis. Survival time without disease progression (yellow bars) and survival time after-progression (brown bars) after CAR-T infusion of individual patients are shown. Patients were divided into 2 groups (CD3^HIGH^ and CD3^LOW^) based on CD3^+^cell counts at leukapheresis. Green dots indicate CD3^+^cell counts at leukapheresis of individual patients. Disease status at infusion [complete response (CR)/partial response (PR) or stable disease (SD)/progressive disease (PD)] of each patient is shown at the bottom.

The clinical courses of all patients are summarized in Fig. 2 and detailed disease status at apheresis and CAR-T infusion, and post-apheresis and lymphocyte depletion therapy are shown in Supplementary Table 3. The cause of death was mainly disease relapse, except for 2 cases with myelodysplastic syndrome (No. 2) and severe cytokine release syndrome (No. 44). In the CD3^HIGH^ group, only one patient of 10 in CR/PR (10.0%) at infusion relapsed after CAR-T cell infusion, while four patients of five in SD/PD (80.0%) relapsed after infusion. In the CD3^LOW^ group, the proportions of patients who experienced disease progression after infusion were similar in CR/PR and SD/PD [2 of 4 patients in CR/PR (50.0%); 7 of 16 in SD/PD (43.8%)]. Interestingly, regarding Hans cell-of-origin classification, among 28 patients in SD/PD at infusion, patients with GCB-phenotypes responded more favorably than those with non-GCB-phenotypes [10 of 14 patients (76.9%) vs. 3 of 14 patients (23.1%), P = 0.021]. Such a difference regarding the classification was not observed in patients treated in CR/PR at infusion.

### Impact of CD3^+^ cell count at leukapheresis on lymphocyte expansion after CAR-T cell infusion

**Figure 3:**
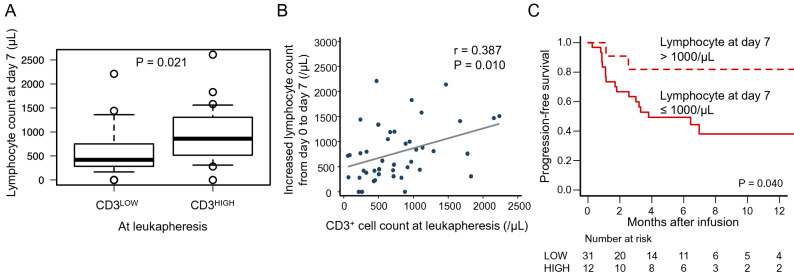
Correlation of lymphocyte counts after CAR-T cell infusion with CD3^+^ cell counts at leukapheresis and their prognostic significance. Box plot showing lymphocyte count at day 7 after infusion in CD3^LOW^ and CD3^HIGH^ group patients (**A**). Scatter plot showing the correlation with CD3^+^ cell counts in peripheral blood at leukapheresis and lymphocyte counts in peripheral blood at day 7 after infusion (**B**). Kaplan–Meier estimates of OS according to lymphocyte count at day 7 (> 1000/μL or ≤ 1000/μL) (**C**).

Because CD3^+^ cell count in peripheral blood at leukapheresis may reflect the function of CAR-T cells, we sought to analyze effects of CD3^+^ cell count at leukapheresis on lymphocyte expansion after infusion of CAR-T cells. Lymphocyte counts in peripheral blood at day 7 were significantly higher in the CD3^HIGH^ group (median 860/μL, range 0–2610/μL) than in the CD3^LOW^ group (median 420/μL, range 0–2210/μL; P = 0.021; Fig. 3A), although lymphocyte count at day 0 and number of infused CAR-T cells were comparable between the two groups. Furthermore, CD3^+^ cell counts in peripheral blood at leukapheresis showed a significantly positive correlation with increased lymphocyte counts from day 0 to day 7 after infusion (P = 0.010) (Fig. 3B). Other blood count data after CAR-T cell infusion in each group are shown in Supplementary Table 4.

Knowing the relationship between CD3^+^ cell count at leukapheresis and lymphocyte increase after infusion, the next step was to analyze the impact of lymphocyte count at day 7 on PFS. 1‐year PFS in patients with lymphocyte counts at day 7 > 1000/μL (81.8%, 95% CI 44.7–95.1%) was significantly higher than in patients with day 7 counts ≤ 1000/μL (38.0%, 95% CI 19.1–56.8%, P = 0.040) (Fig. 3C). The superiority of PFS observed in patients with lymphocyte counts at day 7 > 1000/μL remained after adjustment for disease status at the time of CAR-T cell infusion, though not statistically significant (P = 0.094). These results suggest that proliferation of CAR-T cells after infusion predicted by higher CD3^+^ cell counts at leukapheresis can also be a prognostic marker for outcomes following CAR-T cell therapy.

## Discussion

This study evaluated prognostic impact of CD3^+^ cell counts in peripheral blood at leukapheresis in patients with r/r DLBCL who later received CAR-T cell therapy. There were two major findings regarding CD3^+^ cell count in peripheral blood at leukapheresis: (1) greater CD3^+^ cell numbers resulted in better PFS and OS, and (2) higher CD3^+^ cell counts at leukapheresis were associated with significantly higher lymphocyte counts at day 7, suggesting expansion of CAR-T cells after infusion. Our results indicate that CD3^+^ cell counts in peripheral blood at leukapheresis are useful for predicting outcomes and for designing risk‐adapted strategies including CAR-T cell therapy. The novelty of this study is that it revealed the contribution of parameters on the CAR-T cell side, independent of tumor-intrinsic factors, to the outcome of CAR-T cell therapy.

The positive association of CD3^+^ cell count at leukapheresis and efficacy of CAR-T cell therapy may be driven by at least three factors, including quality of T cells, efficacy of the CAR-T cell manufacturing process, and in vivo environment^13^. First, decreased CD3^+^ cell count in peripheral blood, namely a “low quantity of T cells”, can be a surrogate marker of “inferior T-cell quality” as a source of CAR-T cells. In patients with r/r DLBCL, quality of T cells can be impaired by various factors, such as prolonged dysfunction of T cells caused by an increasing number of chemotherapy regimens^14–16^. In this study, CD3^LOW^ group patients tended to have a higher number of chemotherapy lines than CD3^HIGH^ group patients, although the difference was not statistically significant (Table 1).

Second, although collection of sufficient number of CD3^+^ cells can be achieved via leukapheresis by optimizing leukapheresis procedures even in patients with low CD3^+^ cell counts^17^, subsequent ex vivo manufacturing process of CAR-T cells, where T cells are activated, transduced with CAR, and then expanded, can be potentially complicated in patients with low CD3^+^ cell counts at leukapheresis. Actually, in our cohort, transduction efficiency of CAR was significantly lower (P = 0.02), and CAR expression tended to be lower (P = 0.085) in the CD3^LOW^ group, compared to the CD3^HIGH^ group (Supplemental Table 1). Our findings support previous studies suggesting that efficiency of CAR integration and expression level is low if the leukapheresis product contains T cells with poor proliferative potential^18^.

Third, patients with low CD3^+^ cell counts at leukapheresis, who often have longer history of chemotherapies and more refractory lymphoma, may potentially have disadvantageous in vivo environments for growth and efficacy of infused CAR-T cells. A high tumor burden is one of the factors that degrade the environment, leading to higher immune dysregulation with increased serum inflammatory cytokines and tumor interferon signaling, which results in poor CAR-T cell expansion^19,20^.

Thus, CD3^+^ cell counts at leukapheresis can be a surrogate marker for quality of T cells, efficacy of CAR-T cell production, and in vivo environment. In addition to that, we showed for the first time that CD3^+^ cell counts can predict the number of lymphocytes at day 7 after infusion, suggesting the degree of CAR-T cell expansion. In previous studies, in vivo expansion of CAR-T cells after infusion was reported as a vital marker for function of CAR-T cells^3,21–23^, and early increases in lymphocyte counts are useful for estimating treatment responses. However, there is no established method for evaluating the ability of T cells to generate a CAR-mediated anti-tumor response and for predicting in vivo CAR-T cell expansion, so our finding that CD3^+^ cell counts at leukapheresis predict later expansion will be useful to develop treatment strategies, including CAR-T cell therapy.

Based on these results, comprehensive therapeutic strategies are needed during the entire course of CAR-T cell therapy in order to maximize therapeutic effects of CAR-T cells. Since reduced CD3^+^ cell counts in peripheral blood, which are often caused by repeated, heavy exposure to chemotherapeutic agents, are a marker for degraded quality of the source of CAR-T cells, T cells should be harvested in a way that fully maintains their functions. In Japan, CAR-T therapy is approved for patients refractory to more than two lines of chemotherapy. However, T-cell harvesting are considered before starting intensive salvage chemotherapy, rather than after sufficient washout periods during or after chemotherapy. Especially for a subset of patients who are expected to be at high risk of future relapse or resistance to conventional therapies, it may be beneficial to preemptively harvest and store high-quality T cells before T cells become exhausted, even without stringently meeting the indication for CAR-T cell therapy. T cells from patients early after diagnosis yield greater expansion of CAR-T cells than those from patients with r/r disease^24^. These strategies can enhance CAR-T cell function independent of tumor-intrinsic factors and can improve overall treatment efficiency.

This study has several limitations. First, this is a single-center retrospective analysis with a small number of patients and short follow-up time due to the novelty of CAR-T cell therapy, although our center performs more CAR-T cell therapies than most institutions in Japan and eligibility for CAR-T cell therapy is fairly consistent. In this study, effects of disease status and prior lines of chemotherapies at leukapheresis were carefully adjusted in the multivariate analysis evaluating the impact of CD3^+^ cell count at leukapheresis on survival; however, potential effects of factors associated with tumor intrinsic factors on survival should be further studied in a larger cohort, given the longer time from diagnosis to leukapheresis observed in the CD3^HIGH^ group compared with the CD3^LOW^ group, the different effects of CD^3+^ T cells number on survival according to the cell of origin (data not shown) and the higher response rate observed in patients with GCB-phenotypes compared with non-GCB-phenotypes when disease status was SD or PD at infusion. Second, we did not assess expansion of CAR-T cells themselves or detailed phenotypes of CD3^+^ cells after infusion, while most circulating lymphocytes in the first few weeks after CAR-T cell infusion comprise CAR-T cells themselves^22,23^. In addition, large numbers of T cells without CAR were also suggested to be activated after CAR-T infusion^25^. Therefore, we consider that evaluating CD^3+^ cells, which include both CAR-T cells and non-CAR-T cells, are reasonable to assess the entire effect of CAR-T therapy. Phenotypic differences among CD^3+^ cells are important factors that contributes the expansion of CAR-T cells and the effect of CAR-T therapy. We found that CD4/CD8 ratio at apheresis showed a positive correlation with increased lymphocyte count at day 7 especially in the CD3^LOW^ group (data not shown). Furthermore, previous studies demonstrated that CAR T cell products generated from naïve/stem memory T cells, as compared with unselected T cells, shows superior antitumor activity and increase expansion rates^26,27^. However, because it is difficult to evaluate theses phenotypes in the clinical setting, we used lymphocyte counts in peripheral blood as a simple biomarker reflecting CAR-T cell proliferation^28,29^. Detailed cytokine or chemokine assays were not performed.

In conclusion, we demonstrated that CD3^+^ cell counts at leukapheresis predicts expansion of CAR-T cells after infusion and the overall outcomes of CAR-T cell therapy. Optimizing CAR-T cell therapy at the time of leukapheresis, especially for high-risk patients, should be considered to achieve favorable CAR-T cell responses.

## Supplementary Information

Supplementary Tables.

## Data Availability

The datasets used and/or analyzed during the current study are available from the corresponding author on reasonable request.
