# Supplementary material for: T-cell counts in peripheral blood at leukapheresis predict responses to subsequent CAR-T cell therapy
Source: Sci Rep. 2022 Nov 4;12:18696. doi: 10.1038/s41598-022-23589-9 (PMC9636390; doi:10.1038/s41598-022-23589-9)
Supplement: Supplementary file 1 — Supplementary Tables. [file 41598_2022_23589_MOESM1_ESM.pdf]

Supplemental Table 1. CAR-T cells products' characteristics

	Total (n = 44)	$CD3^{LOW}$ (n = 20)	$CD3^{HIGH}$ (n = 24)	P
Transduction efficiency by CAR qPCR (copies/cell)				0.020
Median (range)	0.47 (0.14–1.08)	0.42 (0.2–0.99)	0.57 (0.14–1.08)	
Viable T cells (%)				0.433
Median (range)	99.5 (98.3–99.9)	99.6 (98.8–99.9)	99.5 (98.3–99.7)	
CAR expression by flow cytometry (%)				0.085
Median (range)	23.2 (7.8–41.4)	21.1 (11.2–36.0)	25.9 (7.8–41.4)	
Release of IFNγ in response to CD19 expressing target cells (fg/transduced cell)				0.724
Median (range)	80 (16–388)	80 (37–346)	78 (16–388)	
Infused CAR-T cell number (10^8)				0.390
Median (range)	6.67 (0.71–12.5)	7.41 (0.71–12.5)	5.84 (0.82–12.1)	

Abbreviations: CAR, chimeric antigen receptor; IFN, interferon

Supplementary Table 2. CRS characteristics according to the number of $CD3^{+}$ cells at leukapheresis.

	Total (n = 44)	$CD3^{LOW}$ (n = 20)	$CD3^{HIGH}$ (n = 24)	P
CRS				1.000
Yes	41 (93.2)	19 (95.0)	22 (91.7)	
No	3 (6.8)	1 (5.0)	2 (8.3)	
CRS grade				0.366
1	32 (78.0)	13 (68.4)	19 (86.4)	
2	7 (17.1)	5 (26.3)	2 (9.1)	
3	2 (4.9)	1 (5.3)	1 (4.5)	
Tocilizumab administration				1.000
Yes	27 (61.4)	12 (60.0)	15 (62.5)	
No	17 (38.6)	8 (40.0)	9 (37.5)	

Abbreviations: CRS, cytokine release syndrome

Supplementary Table 3. Disease status at apheresis and CAR-T infusion, and post-apheresis and lymphocyte depletion therapy according to the number of $CD3^{+}$cells at leukapheresis.

	Total (n = 44)	$CD3^{LOW}$ (n = 20)	$CD3^{HIGH}$ (n = 24)	P
Disease status at apheresis				0.697
CR	1 (2.3)	0 (0.0)	1 (4.2)	
PR	13 (29.5)	5 (25.0)	8 (33.3)	
SD	20 (45.5)	11 (55.0)	9 (37.5)	
PD	10 (22.7)	4 (20.0)	6 (25.0)	
Bridging chemotherapy				0.966
Gem based chemotherapy	16 (36.4)	7 (35.0)	9 (37.5)	
Other intensive chemotherapy	18 (40.9)	9 (45.0)	9 (37.5)	
Reduced intensity therapy	3 (6.8)	1 (5.0)	2 (8.3)	
No	7 (15.9)	3 (15.0)	4 (16.7)	
Lymphocyte depletion chemotherapy				0.356
FluCy	37 (84.1)	18 (90.0)	19 (79.2)	
Bendamustine based	5 (11.4)	1 (5.0)	4 (16.7)	
Others	1 (2.3)	1 (5.0)	0 (0.0)	
No	1 (2.3)	0 (0.0)	1 (4.2)	
Disease status at infusion				0.217
CR	4 (9.1)	0 (0.0)	4 (16.7)	
PR	11 (25.0)	4 (20.0)	7 (29.2)	
SD	12 (27.3)	7 (35.0)	5 (20.8)	
PD	17 (38.1)	9 (45.0)	8 (33.3)	

Abbreviations: CR, complete response; PR, partial response; SD, stable disease; PD, progressive disease; Gem, Gemcitabine; FluCy, Fludarabine and Cyclophosphamide.

Supplementary Table 4. Blood count after CAR-T cell infusion according to the number of $CD3^{+}$cells at leukapheresis (median, range).

	Total (n = 44)	$CD3^{LOW}$ (n = 20)	$CD3^{HIGH}$ (n = 24)	P
WBC (10^9/L)				
Day 0	1.72 (0.07-7.43)	1.42 (0.07-5.57)	1.84 (0.33-7.43)	0.444
Day 7	1.94 (0.15-16.06)	1.42 (0.15-7.97)	2.36 (0.36-16.06)	0.115
Day 14	2.16 (0.62-6.62)	2.16 (0.62-6.62)	2.19 (0.60-6.18)	0.929
Hb (g/dL)				
Day 0	8.65 (6.70-12.70)	8.55 (6.70-12.70)	9.65 (6.70-12.50)	0.345
Day 7	8.50 (6.50-11.40)	8.10 (7.10-10.90)	8.65 (6.50-11.40)	0.155
Day 14	9.35 (6.20-12.20)	8.95 (6.20-12.20)	9.70 (6.50-12.10)	0.098
Plt (10^9/L)				
Day 0	124.00 (7.00-327.00)	118.50 (7.00-265.00)	132.00 (18.00-327.00)	0.786
Day 7	99.00 (16.00-249.00)	99.00 (16.00-226.00)	101.50 (33.00-249.00)	0.990
Day 14	107.50 (23.00-406.00)	106.00 (23.00-406.00)	121.00 (25.00-279.00)	0.741

Abbreviations: WBC, white blood cell; Hb, hemoglobin; Plt, platelet.
